# Supplementary material for: Analysis of PPARγ Signaling Activity in Psoriasis
Source: Int J Mol Sci. 2021 Aug 10;22(16):8603. doi: 10.3390/ijms22168603 (PMC8395241; doi:10.3390/ijms22168603)
Supplement: Supplementary file 1 [file ijms-22-08603-s001.zip › Supplemental materials_Analysis of PPARg signaling activity in psoriasis/Pathway models/Models images and html files/Interleukin-17 and interleukin-22 signaling in psoriasis/179382.html]

PIP3


# Small Molecule PIP3

|  |  |
| --- | --- |
| URN | urn:agi-cas:165689-82-7 |
| Total Entities | 0 |
| Connectivity | 1339 |
| Name | PIP3 |
| Description | phosphatidylinositol-triphosphate |
| Class | Endogenous compound |
| ObjectType | Small Molecule |

---

|  |  |
| --- | --- |
| Pathway | Osteoarthritis |
|  | Androgen Deficiency in Male Obesity |
|  | Oncogene Induced Cellular Senescence |
|  | MTHFR Activity Regulation |
|  | DNA Persistent Repair Inhibits mTOR Signaling |
|  | mTOR Signaling Activation by Fatty Acids and Glucose |
|  | mTOR Signaling Activation by Amino Acids |
|  | mTOR Signaling |
|  | mTOR Signaling Hyperfunction |
|  | Insulin Influence on Protein Synthesis |
|  | Insulin Influence on Glycogenesis |
|  | Insulin Influence on Lipogenesis |
|  | Dopamine Receptors Signaling |
|  | Serotonin Receptors Signaling |
|  | Thyroid Hormones Common non-Genomic Effects in Hyperthyroidism |
|  | Insulin Signaling |
|  | Prolactin Production |
|  | Thrombopoietin Receptors Signaling in Platelet Maturation |
|  | Androgens in Adipocyte Activation |
|  | LH and FSH Signaling and Steroidogenesis |
|  | Toll-like Receptors Act through MYD88-TIRAP Signaling |
|  | T-Cell Dependent B-Cell Activation |
|  | T-Cell Independent B-Cell Activation |
|  | Natural Killer Cell Activation through C-Type Lectin-like Receptors |
|  | Natural Killer Cell Activation through ITAM-Containing Receptors |
|  | Natural Killer Cell Inhibitory Receptor Signaling |
|  | Natural Killer Cell Activation through ITSM-Containing Receptors |
|  | Natural Killer Cell Activation through Integrins and non-ITAM-Containing Receptors |
|  | T-Cell Maturation (Hypothesis) |
|  | Natural Killer Cell Activation |
|  | Peripheral T-Cell Tolerance |
|  | T-Cell Positive Selection and Neglect Induced Death |
|  | T-Cell Central Tolerance |
|  | CD8+ T-Cell Activation |
|  | Th1-Cell Differentiation |
|  | Treg-Cell Differentiation |
|  | Th2-Cell Differentiation |
|  | T-Cell Receptor Signaling |
|  | Th17-Cell Differentiation |
|  | AHR Signaling in Tr1 Cells Function |
|  | CXC Chemokine Receptor Signaling |
|  | CC Chemokine Receptor Signaling |
|  | Plasmin Effects in Inflammation |
|  | TAM Receptors in Platelet Aggregation |
|  | Platelet Activation via Adhesion Molecules |
|  | Scavenger Receptors in Platelet Aggregation |
|  | Proplatelet Maturation |
|  | Bradykinin Effects in Inflammation |
|  | Platelet Activation via GPCR Signaling |
|  | Toll-like Receptors in Sterile Inflammation |
|  | Toll-like Receptor Independent Sterile Inflammation |
|  | Lipoxin A4/FPR2-Related Neutrophil Depression |
|  | Annexins in Inflammation Resolution |
|  | Eat me Signal: Apoptotic Cell Induces Phagocytosis |
|  | Eicosanoids in Myeloid Cell Activation |
|  | Histamine Receptor H3 Signaling in Neurons |
|  | Neutrophil Activation via LTB4/Polyisoprenyl Phosphate Signaling |
|  | Basophil Activation |
|  | Eosinophil Survival by Cytokine Signaling |
|  | Eosinophil Activation |
|  | Mast-Cell Activation via IgE Signaling |
|  | Mast-Cell Activation without Degranulation through CYSLTR1/CYSLTR2 Signaling |
|  | Mast-Cell Activation without Degranulation |
|  | Mast-Cell Activation without Degranulation through KIT/F2R Signaling |
|  | Mast-Cells De Novo Synthesized Mediators via IgE Independent Signaling |
|  | CD16/CD14 Proinflammatory Monocyte Activation |
|  | Macrophage M2-Related Phagocytosis |
|  | anti-Inflammatory Function of Macrophage M2 Lineage |
|  | Neutrophil Activation via FCGR3B |
|  | Neutrophil Degranulation via FPR1 Signaling |
|  | CR3 Mediated Phagocytosis in Neutrophils and Macrophages |
|  | Neutrophil Chemotaxis |
|  | Neutrophil Activation via Adherence on Endothelial Cells |
|  | VEGFA/NOTCH1/WNT Cross-talk in Blood Vessel Sprouting and Branching in Cancer |
|  | Vascular Endothelial Cell Activation by NO |
|  | Vascular Smooth Muscle Cell/Pericyte Migration and Proliferation |
|  | Vascular Endothelial Cell Activation by Cytokines |
|  | Vascular Endothelial Cell Activation by Blood Coagulation Factors |
|  | Vascular Endothelial Cell Activation by Growth Factors |
|  | Vascular Endothelial Cell Permeability Activation |
|  | Adipokines Production by Adipocyte |
|  | Lipogenesis Regulation in Adipocyte |
|  | Synaptic Potentiation by PKMZ in LTP Maintenance - Active State |
|  | Potassium Flux Regulation |
|  | GFs/TNF -> Ion Channels |
|  | Nociception Expression Targets Signaling |
|  | Sodium Flux Regulation |
|  | Vascular Motility |
|  | Ca2+ Flux Regulation |
|  | NPY1R -> CRH/POMC Production |
|  | NTSR1 Expression Targets |
|  | BDKRB1/2 -> Vasodilation |
|  | BDKRB1/2 -> Interleukins Production |
|  | CNR1/2 -> Membrane Transport |
|  | CNR1/2 -> IL1B/2/4/6/10 Production |
|  | DRD2 Expression Targets |
|  | DRD2 -> TRPC1 Transcription |
|  | DRD2/4 -> Membrane Transport |
|  | GALR1/2/3 -> POMC/NPY Production |
|  | CHRM1 -> IL2 Production |
|  | CHRM1/2/3/5 -> Ion Channels |
|  | PTGER2/3 -> Vascular Motility |
|  | HTR1 -> IL6 Production |
|  | HTR5 -> TNF Production |
|  | HTR1 -> Membrane Transport |
|  | TACR1 -> TNF/IL6/IL8 Production |
|  | Leptin -> NO Production/Vasodilation |
|  | NTRK1/2/3 -> Acetylcholine Production |
|  | EPHB -> NMDA Receptor Activation |
|  | non-Genomic Rapid Actions of Vitamin D in Vitamin D Biology |
|  | G0/G1 Phase Transition |
|  | Lamellipodia and Filopodia Formation in Cancer |
|  | RAGE/AGER and S100 Proteins in Cardiovascular Injury |
|  | Th1-Cells Activation and Proliferation in Atherosclerosis |
|  | Dilated Cardiomyopathy |
|  | Cardiomyocyte Hypertrophy |
|  | Hypertrophic Cardiomyopathy |
|  | Endothelin Signaling in Arterial Hypertension |
|  | Arterial Hypertension |
|  | Endothelial Cell Dysfunction in Arterial Hypertension |
|  | Smooth Muscle Cell Dysfunction in Arterial Hypertension |
|  | TRPM4/6/7/8 Signaling in Arterial Hypertension (Hypothesis) |
|  | Smooth Muscle Cell Dysfunction in Pulmonary Hypertension |
|  | Myocardial Remodeling |
|  | Myocardial Infarction |
|  | Preconditioning Ischemia |
|  | Lymphocyte Mediated Myocardial Injury in Myocarditis |
|  | Myocarditis, Acute Viral Overiew |
|  | Vascular Reactivity in Raynaud Disease |
|  | NO Synthesis in Early Vasospasm |
|  | Neurons Necrosis Caused by Energy Deficiency |
|  | Contraction Due Vasospasm |
|  | Hyperseborrhea in Acne Vulgaris |
|  | Androgen Induced Sebocyte Hyperfunction |
|  | Androgens in Sebocyte Maturation |
|  | Atopic Dermatitis Onset |
|  | Cell Cycle Dysregulation in Melanoma |
|  | Melanoma |
|  | Apoptosis Block in Melanoma Cell |
|  | Dedifferentiation and Metastatic Progression in Melanoma |
|  | MITF as a Regulator of Melanoma Cell Development |
|  | IFN-gamma/TNF-alpha Mediated Cell Proliferation in Psoriasis |
|  | IL22 Induces Keratinocyte Proliferation in Psoriasis |
|  | Dendritic Cells Function in Psoriasis |
|  | Apoptotic Keratinocytes Clearance Recession in Systemic Lupus Erythematosus |
|  | Th0-Cell Aberrant Activation in Systemic Lupus Erythematosus |
|  | Th1-Cell Function in Systemic Lupus Erythematosus |
|  | B-Cell Activation in Systemic Lupus Erythematosus |
|  | Th2-Cell Function in Systemic Lupus Erythematosus |
|  | Oxidative Stress in Vitiligo |
|  | Acute Cytotoxic CD8+T-Cell Response against Melanocytes |
|  | Vitiligo |
|  | T-Cell Cytotoxic Response against Melanocytes in Vitiligo |
|  | Apoptosis of Melanocyte in Vitiligo |
|  | Humoral Immunity in Vitiligo |
|  | Treg-Cell Function in Diabetes Mellitus Type 1 |
|  | Natural Killer T-Cell Roles in Diabetes Mellitus Type 1 |
|  | Natural Killer Cell in Diabetes Mellitus Type 1 |
|  | Toll-like Receptors in beta-Cell |
|  | Macrophage Function in Diabetes Mellitus Type 1 |
|  | Treg-Cell Activation in Diabetes Mellitus |
|  | Donohue Syndrome Progression (Hypothesis) |
|  | Advanced Glycation End Products Pathway in Diabetic Microangiopathy |
|  | Peripheral Tissue Microangiopathy |
|  | Tubular Cell Dysfunction in Progressive Diabetic Nephropathy |
|  | Podocyte Dysfunction in Diabetic Nephropathy |
|  | Endothelial Cell Dysfunction in Early Diabetic Nephropathy |
|  | Mesangial Cell Dysfunction in Diabetic Nephropathy |
|  | Microangiopathy in Diabetic Neuropathy |
|  | Proliferative Diabetic Retinopathy |
|  | Glucocorticoids in Insulin Resistance in Skeletal Muscles |
|  | Adipokines and Cytokines in Insulin Resistance in Skeletal Muscles |
|  | AGE Related Insulin Resistance in Skeletal Muscles |
|  | Adiponectin Synthesis Declines in Insulin Resistance |
|  | Gluconeogenesis Impairment in non-Alcoholic Fatty Liver Disease |
|  | PNPLA3 in non-Alcoholic Fatty Liver Disease |
|  | Lipid Metabolism Impairement in non-Alcoholic Fatty Liver Disease |
|  | Leptin in Insulin Synthesis and Secretion |
|  | beta-Cell Destruction through Cytokines in Diabetes Mellitus |
|  | beta-Cell Mass Regulation |
|  | beta-Cell Function Inhibition by Ciclosporine and Tacrolimus (Rodent Model) |
|  | FOXO1 and SREBP-1C Role in beta-Cell Suppression (Rodent Model) |
|  | RET1 Mutation in Hyperparathyroidism |
|  | Bone Remodeling in Hyperthyroidism |
|  | Cardiovascular Effects in Hyperthyroidism |
|  | Thyroid Dysfunction |
|  | Graves Ophthalmopathy |
|  | Immune System Activation in Hyperthyroidism |
|  | Hypothyroidism, Secondary (Central) |
|  | Thyroid Stimulating Hormone (TSH) Resistance in Congenital Hypothyroidism |
|  | Overt Hypothyroidism, Primary |
|  | Thyroid Stimulating Hormone (TSH) Secretion in Overt Hypothyroidism |
|  | Thymic Follicular Hyperplasia |
|  | Adipocyte Hypertrophy and Hyperplasia |
|  | Adipokines Production by Adipocyte Impaired in Obesity |
|  | Prolactinoma Overiew |
|  | CD4+T-Cell Response in Celiac Disease |
|  | Enterocyte Mediated Mucosal Damage in Celiac Disease |
|  | CD8+ T-Cell Response in Celiac Disease |
|  | Metastatic Colorectal Cancer |
|  | GUCY2C Signaling in Colorectal Cancer |
|  | OipA Signaling in Helicobacter Pylori Infection |
|  | CagA Phosphorylation Dependent Signaling |
|  | EDN, NRG, NRTN, and GDNF/RET Signaling in Hirschsprung Disease |
|  | Peutz-Jeghers Syndrome |
|  | Dendritic Cell Function in Ulcerative Colitis |
|  | Nicotine anti-Inflammatory Effect in Ulcerative Colitis |
|  | Th2-Cell Function in Ulcerative Colitis |
|  | T-Cell Cytotoxic Response in Ulcerative Colitis |
|  | FLT3/KIT/MLL Signaling in Leukemia |
|  | Apoptosis Block in Acute Myeloid Leukemia Cell |
|  | RARA Signaling in Acute Myeloid Leukemia (M3) |
|  | Acute Myeloid Leukemia |
|  | Antiphospholipid Antibodies in Platelets and Coagulation Cascade |
|  | B-Cell Acute Lymphoblastic Leukemia |
|  | B-Cell Chronic Lymphocytic Leukemia |
|  | Chronic Myeloid Leukemia |
|  | Diffuse Large-B-Cell Lymphoma |
|  | Diffuse Large-B-Cell Lymphoma ABC Subtype |
|  | Lymphoma, GCB Subtype |
|  | Follicular Lymphoma |
|  | Hodgkin Lymphoma |
|  | Mantle Cell Lymphoma |
|  | mTOR/NF-kB/BCR Signaling Disregulation in Mantle Cell Lymphoma |
|  | Hedgehog Signaling in Mantle Cell Lymphoma |
|  | Mucosa-Associated Lymphoid Tissue (MALT) Lymphoma |
|  | Osteoclast Activation in Multiple Myeloma |
|  | IL6/IGF1/VEGFA Signaling in Multiple Myeloma |
|  | Multiple Myeloma |
|  | Platelet and Coagulation Cascade Activation by Antiphospholipid Antibodies |
|  | T-Cell Acute Lymphoblastic Leukemia |
|  | Hepatocellular Carcinoma |
|  | Growth Factor Signaling in Hepatocellular Carcinoma |
|  | Pancreatic Ductal Carcinoma |
|  | Growth Factor Signaling in Pancreatic Neoplasms |
|  | Pancreatic Neuroendocrine Tumors |
|  | STK11 Signaling in Pancreatic Neoplasms |
|  | Il17 Signaling Related Neutrophilia in Asthma |
|  | Eosinophil Activation and Degranulation in Asthma |
|  | Airway Smooth Muscle Cell High Rate Proliferation |
|  | IgE Induces Airway Smooth Muscle Cell Proliferation |
|  | Goblet-Cell Exocytosis in Asthma |
|  | Goblet-Cell Related Mucus Secretion in Asthma |
|  | Neutrophil Degranulation via FPR1/IL8 |
|  | Basophil Activation in Asthma |
|  | Mast-Cell Activation in Asthma |
|  | Mast-Cell Related Chemokines Signaling in Asthma |
|  | B-Cell Mediated IgE Production in Asthma |
|  | Th17-Cell Differentiation in Asthma |
|  | Th2-Cell Response in Asthma |
|  | Ovulation Block |
|  | SHBG and IGFBP1 Synthesis Decline in Polycystic Ovary Syndrome |
|  | Polycystic Ovary Syndrome |
|  | FSH Action in Polycystic Ovary Syndrome |
|  | Dendritic Cell Dysfunction in Psoriatic Arthritis |
|  | Th17-Cell and Th1 Immune Responsein Psoriatic Arthritis |
|  | Keratinocyte Activation in Psoriatic Arthritis |
|  | B-Cells Function in Systemic Scleroderma |
|  | Th2-Cells Function in Systemic Scleroderma |
|  | CD4+ T-Cell Function Decline in HIV |
|  | Macrophage Survival through CCR5 and CXCR4 Mediated Signaling |
|  | Toll-like Receptors (TLR) in Periodontitis |
|  | Diabetes Induced Periodontitis |
|  | Estrogen Effects on Pregnancy Gingivitis |
|  | IL1 Receptor Antagonist (IL1RN) Deficiency (DIRA) |
|  | Mevalonate Kinase Deficiency |
|  | Blau Syndrome |
|  | Familial Cold Autoinflammatory Syndrome 2 |
|  | Hereditary Hemorrhagic Telangiectasia |
|  | Cowden Syndrome |
|  | Glutamate Toxiicity Effects on Striatum Medium Spiny Neurons in Hantington's Disease |
|  | IL6 and Insulin Resistance in Muscular Dystrophies |
|  | Ca2+ Overload in Muscular Dystrophies |
|  | Muscular Dystrophies |
|  | Dystrophin Glycoprotein Complex Signaling in Muscular Dystrophies |
|  | IGF1 Role in Muscle Hypertrophy |
|  | Myostatin-IGF1 Crosstalk in Skeletal Muscles in Muscular Dystrophies |
|  | Osteoblast Function Decline in Osteoporosis |
|  | BDNF Upregulation Triggered by Seizures |
|  | Ketogenic Diet Mechanism (Hypothesis) |
|  | Blood-Brain Barrier Disruption in Epileptiform Disorders |
|  | mTOR Hyperactivation after Status Epilepticus |
|  | Glioblastoma, Primary |
|  | Glioblastoma, Secondary |
|  | Astrocytoma |
|  | Glioma Invasion Signaling |
|  | Glioma Stem Cell Program Activation |
|  | Ependymoma |
|  | Medulloblastoma |
|  | Vascular Endothelial Cell Vasodilation in Brain |
|  | Dopamine Action in CSD Progression |
|  | Postsynaptic Neuron Activation |
|  | nNOS (NOS1) and iNOS (NOS2) Activation via Glutamate |
|  | Growth Factor Signaling in Neuroblastoma |
|  | Neuroblastoma |
|  | ALK Associated Neuroblastoma |
|  | Glutamate Role in Parkinson's Disease |
|  | Glutamate Mediated Excitotoxicity in Amyotrophic Lateral Sclerosis |
|  | Microglia and Motor Neuron Interaction Dysregulation |
|  | MIR155 Role in Th17-Cell in Multiple Sclerosis |
|  | SPP1 Induced Survival of Myelin Specific T-Cells in Multiple Sclerosis |
|  | Brain Cell Necrosis in Vascular Dementia |
|  | ERBB/VEGFR/Akt Signaling in Breast Cancer |
|  | IGF1R/AKT Signaling in Breast Cancer |
|  | ESR1/ERBB Positive Luminal Breast Cancer |
|  | Breast Cancer |
|  | ESR1 Signaling in Breast Cancer |
|  | Endometrial Cancer |
|  | Endometrioid Endometrial Cancer |
|  | Clear Cell Endometrial Cancer and Papillary Serous Endometrial Cancer |
|  | Angiogenesis in Endometriosis |
|  | Coagulation Factors and PAR1/2 Receptors (F2R/F2RL1) in Endometriosis |
|  | Endometrioid Ovarian Carcinoma |
|  | Clear Cell Ovarian Carcinoma |
|  | High-Grade Serous Ovarian Carcinoma |
|  | Low-Grade Serous Ovarian Carcinoma |
|  | Ovarian Cancer |
|  | Mucinous Ovarian Carcinoma |
|  | LH and FSH Signaling Impairment in Primary Ovarian Insufficiency |
|  | FOXO3 and PTEN Inactivation in Premature Ovarian Failure (Mouse Model) |
|  | MIF Role in Cholesteatoma |
|  | EGF/cyclinD1 Signaling in Acquired Middle Ear Cholesteatoma |
|  | HMGB1/RAGE Signaling in Middle Ear Cholesteatoma |
|  | Cholesteatoma |
|  | HPV E5 Regulates EDNRA and EGFR Signaling Pathway in Keratinocytes |
|  | Androgen Receptor/Akt Signaling |
|  | Prostate Cancer |
|  | Androgen Receptor/SGK1 Signaling |
|  | EPHA2 Mutations in Cataract |
|  | Steroids Induced Cataract |
|  | VEGFA in Neovascular Glaucoma |
|  | Glutamate Mediated Excitotoxicity in Glaucoma |
|  | Neurotrophic Factor Deprivation in Retinal Ganglion Cell Death |
|  | Immune System Role in Otitis Media |
|  | Alveolar Macrophages Dysfunction in COPD |
|  | T-Cell Cytotoxic Activation |
|  | Alveolar Epithelial Cell Dysfunction in COPD |
|  | Polycystins Mutation Effects in Cystic Kidney Disease |
|  | Penile Detumescense Augmentation Mechanisms |
|  | Advanced Glycation End Products in Diabetes or Age Related Erectile Dysfunction |
|  | Plasmin Role in Glomerulonephritis |
|  | Endothelial Cell Dysfunction in Glomerulonephritis |
|  | Ammonia Effects on Brain Cells |
|  | CD157/ITGB2 Signaling in Myeloid Cell |
|  | CD38/CD3 -> JUN/FOS/NF-kB Signaling in T-cell Proliferation |
|  | CD38/CD19 -> JUN/FOS/NF-kB Signaling in B-cell Proliferation |
|  | S100P Expression Target |
|  | HMGB1 Expression Targets |
|  | TTR Expression Targets |
|  | S100B Expression Targets |
|  | ANGPT1 -> CREB/CREBBP Expression Targets |
|  | ANGPT2 -> AP-1/CREBBP/MYC Expression Targets |
|  | AREG -> NFATC Expression Target |
|  | AREG -> CREB/CREBBP Expression Targets |
|  | AREG -> NCOR2 Expression Targets |
|  | AREG -> HIF1A Expression Targets |
|  | AREG -> AP-1 Expression Targets |
|  | AREG -> FOXO3A Expression Target |
|  | Noradrenaline/Gs Expression Targets |
|  | Epinephrine/Gs Expression Targets |
|  | Acetylcholine Expression Targets |
|  | Dopamine/Gi Expression Targets |
|  | Aryl Hydrocarbon Receptor Genomic and non-Genomic Signaling |
|  | B-Cell Receptor -> NFATC Signaling |
|  | B-Cell Receptor -> AP-1 Signaling |
|  | B-Cell Receptor -> NF-kB Signaling |
|  | BTC -> NFATC Expression Targets |
|  | BTC -> AP-1/ATF/CREB Expression Targets |
|  | BTC -> EP300/ETS/ETV/SP1 Expression Targets |
|  | AGTR1 -> ARRB1/ARRB2 Signaling |
|  | Hedgehog Family -> ARRB1/ARRB2 Canonical Signaling |
|  | ARRB1/ARRB2 non-CanonicalSignaling and HedgehogFamily |
|  | CD19 Expression Targets |
|  | CD81 -> AP-1 Expression Targets |
|  | CD247 Expression Targets |
|  | gamma Globulins Expression Targets |
|  | FCGR3A Expression Targets |
|  | CD22 -> NF-kB Expression Targets |
|  | CD19/CR2 Expression Targets |
|  | CD8 -> AP-1 Expression Targets |
|  | CD72 -> CREB/CREBBP Expression Targets |
|  | CD72 -> NF-kB Expression Targets |
|  | CD72 -> STAT Expression Target |
|  | CD80 -> STAT Expression Targets |
|  | CD81 Expression Targets |
|  | CD80 -> NF-kB Expression Targets |
|  | CD80 -> NFATC Expression Targets |
|  | CD86 -> NFATC Expression Targets |
|  | CD86 -> STAT Expression Targets |
|  | CD86-> NFATC Expression Targets |
|  | CD86 -> NF-kB Expression Targets |
|  | Inositol Phosphate Metabolism |
|  | Tamoxifen Induced Endometrial Cancer |
|  | Dexamethasone Induced Diabetes |
|  | Doxorubicin Induced Cardiotoxicity |
|  | Ritonavir Induced Diabetes |
|  | Clozapine Induced Granulocytopenia |
|  | EGF -> HIF1A Expression Targets |
|  | EGF -> AP-1/ATF Expression Targets |
|  | EGF -> CREB/CREBBP/ELK/SRF/MYC Expression Targets |
|  | EGF -> MEF/MYOD/NFATC Expression Targets |
|  | EGF -> FOXO3A Expression Targets |
|  | EGF -> TP53 Expression Targets |
|  | EGF -> NCOR2 Expression Target |
|  | EGFR -> NCOR2 Signaling |
|  | EGFR/ERBB3 -> MEF/MYOD/NFATC/MYOG Signaling |
|  | EGFR/ERBB2 -> TP53 Signaling |
|  | ERBB2/3 -> EP300/ETS/ETV/SP1 Signaling |
|  | EGFR/ERBB2 -> HIF1A Signaling |
|  | EGFR -> AP-1/ATF2 Signaling |
|  | EGFR -> AP-1/CREB/ELK/SRF/MYC Signaling |
|  | EREG -> CREB Expression Target |
|  | EREG -> EP300/SP1 Expression Targets |
|  | EREG -> FOXO3A Expression Target |
|  | EREG -> HIF1A Expression Target |
|  | EREG -> AP-1/ATF Expression Targets |
|  | Erythropoietin -> FOXO3A Expression Targets |
|  | Erythropoietin -> NF-kB Expression Targets |
|  | Erythropoietin -> AP-1/MYC/CREB Expression Targets |
|  | Erythropoietin -> ELK/SRF Expression Targets |
|  | PECAM1 Expression Targets |
|  | F2 -> AP-1/CREB/ELK/SRF/SP1 Expression Targets |
|  | F2 -> STAT1/NF-kB Expression Targets |
|  | PLG -> AP-1/CREB/ELK/SRF/SP1 Expression Targets |
|  | PLG -> STAT1/NF-kB Expression Targets |
|  | FGF9 -> AP-1/CREB/MYC Expression Targets |
|  | FGF10 -> AP-1/CREB/CREBBP/MYC Expression Targets |
|  | FGF2 -> FOXO3A Expression Targets |
|  | FGF1 -> AP-1/CREB/ELK/SRF/MYC Expression Targets |
|  | FGF1 -> NCOR2 Expression Target |
|  | FGF7 -> FOXO3A Expression Target |
|  | FGF8 -> AP-1/CREB/MYC Expression Targets |
|  | FGF18 -> AP-1/CREB Expression Targets |
|  | FGF23 -> NCOR2 Expression Targets |
|  | FGF10 -> FOXO3A Expression Target |
|  | FGF2 -> NCOR2 Expression Targets |
|  | FGF4 -> AP-1/MYC Expression Targets |
|  | FGF7 -> AP-1/CREB/CREBBP/MYC Expression Targets |
|  | FGF2 -> AP-1/CREB/CREBBP/ELK/SRF/MYC Expression Targets |
|  | FGFR -> AP-1/CREB/CREBBP/ELK/SRF/MYC Signaling |
|  | FPR1 -> Cytoskeleton Signaling |
|  | FSHR Expression Targets |
|  | GH1/GHR -> NF-kB/ELK/SRF/MYC Expression Targets |
|  | CSH1/GHR Expression Targets |
|  | HBEGF -> FOXO3A Expression Target |
|  | HBEGF -> AP-1/ATF Expression Targets |
|  | HBEGF -> TP53 Expression Targets |
|  | HBEGF -> EP300/ETS/ETV/SP1 Expression Targets |
|  | HBEGF -> HIF1A Expression Targets |
|  | HBEGF -> MEF/MYOD Expression Target |
|  | HBEGF -> CREB/MYC Expression Targets |
|  | HGF -> FOXO3A Expression Targets |
|  | HGF -> AP-1/CREB/ELK/SRF/MYC Expression Targets |
|  | HGFR -> FOXO3A Signaling |
|  | HGFR -> AP-1/CREB/MYC Signaling |
|  | TAM Receptor Signaling Activation in Cancer |
|  | PI3K/AKT/MTOR Signaling Activation in Cancer |
|  | G0/G1 Cell Cycle Phase Transition Activation in Cancer |
|  | RAS/RAF/MAPK Signaling Activation in Cancer |
|  | N2 Neutrophils in Tumor-Promoting Inflammation and Tumor Progression |
|  | RAS/RAF/MAPK Signaling Activation by Blocking of Tumor Suppressors |
|  | TGFB Signaling activation by Blocking of Tumor Suppressors |
|  | PI3K/AKT/MTOR Signaling Activation by Blocking of Tumor Suppressors |
|  | Hippo/YAP1 Signaling Deregulation in Cancer |
|  | Hypoxia-Induced Cell Death Evasion in Cancer |
|  | HIF1A in Vasculogenic Mimicry of Cancer |
|  | VEGFA Dependent Angiogenesis in Cancer |
|  | ANGPT/TEK Maintains Endothelial Cell Quiescence in Cancer |
|  | Inhibition of THBS1 Activates Angiogenesis in Cancer |
|  | VEGF Independent Angiogenesis in Cancer |
|  | Integrins in Cancer Cell Motility, Invasion and Survival |
|  | Epithelial to Mesenchymal Transition in Cancer: Overview |
|  | BSG (CD147) in Cancer Cells Motility, Invasion and Survival |
|  | CDH1 Down regulation Promotes Cancer Cell Migration and Metastases |
|  | MTOR/TORC in Cancer Cell Motility and Invasion |
|  | Hyaluronic Acid, CD44 and HMMR in Cancer Cell Invasion and Survival |
|  | CDH2 Activation Promotes Cancer Cell Migration and Survival |
|  | TGFB Family in Epithelial to Mesenchymal Transition in Cancer |
|  | Invadopodia Formation in Cancer Cells |
|  | Skeletal Muscle Wasting in Cancer Cachexia |
|  | Metabolic Effects of Oncogenes and Tumor Suppressor in Cancer Cells |
|  | Lipogenesis/Lipolysis Activation in Cancer Cells and Cancer-Associated Adipocytes |
|  | Treg Cells Promote Immunosuppression in Cancer Immune Escape |
|  | IDO1 in Cancer Immune Escape |
|  | Effector T-cell Inactivation in Cancer Immune Escape |
|  | IGF1 -> MEF/MYOD/MYOG Expression Targets |
|  | IGF1 -> CEBPA/FOXO1A Expression Targets |
|  | IGF2 -> CEBPA/FOXO1A Expression Targets |
|  | IGF2/MEF/MYOD Expression Targets |
|  | Insulin -> CEBPA/CTNNB/FOXA/FOXO Expression Targets |
|  | Insulin -> MEF/MYOD Expression Targets |
|  | Insulin -> ELK/SRF/HIF1A/MYC/SREBF Expression Targets |
|  | CD19 -> NF-kB Signaling |
|  | AGER -> NF-kB Signaling |
|  | CD19 -> JUN/ELK Signaling |
|  | ICAM2 -> CTNNB/FOXO/STAT3 Signaling |
|  | FcIgER -> ELK-SRF Signaling |
|  | InsulinR -> CTNNB/FOXA/FOXO Signaling |
|  | InsulinR -> ELK/SRF/SREBF Signaling |
|  | IGF1R -> CEBPA/FOXO1A Signaling |
|  | IGF1R -> MEF/MYOD/MYOG Signaling |
|  | FibronectinR -> CTNNB Signaling |
|  | FibronectinR -> NF-kB Signaling |
|  | FibronectinR -> AP-1/ELK/SRF/SREBF Signaling |
|  | MacrophageR -> CEBPB -> NF-kB Signaling |
|  | Fibronectin Expression Targets |
|  | CTGF -> AP-1/CREB/MYC Expression Targets |
|  | CTGF -> FOXO3A Expression Target |
|  | CTGF -> NCOR2 Expression Target |
|  | ICAM1 Expression Targets |
|  | Fibrinogen Expression Targets |
|  | IL7R -> FOXO/NF-kB Signaling |
|  | IL15R -> NF-kB/NFATC Signaling |
|  | IL2R -> ELK/SRF/MYC Signaling |
|  | IL23R -> STAT3/NF-kB Signaling |
|  | IL12R -> NF-kB/NFATC Signaling |
|  | IL4R -> ELK/SRF/HMGY Signaling |
|  | IL7 Expression Targets |
|  | IL15 Expression Targets |
|  | IL12B Expression Targets |
|  | IL4 Expression Targets |
|  | IL2 Expression Targets |
|  | UrokinaseR -> ELK/SRF Signaling |
|  | LPA Expression Targets |
|  | PAF/Gq -> AP-1/ATF1/CREB/ERK/SRF Expression Targets |
|  | Dronabinol/Anandamide Expression Targets |
|  | PAF/Gq -> NF-kB Expression Targets |
|  | NRG1 -> MEF/MYOD Expression Targets |
|  | NRG1 -> EP300/ETS/ETV/SP1 Expression Targets |
|  | NRG1 -> CREB/CREBBP/ELK/SRF/MYC Expression Targets |
|  | NRG1 -> FOXO3A Expression Targets |
|  | NRG1 -> TP53 Expression Target |
|  | NRG1 -> AP-1/ATF Expression Targets |
|  | NRG1 -> HIF1A Expression Target |
|  | NGF -> SMAD3/NF-kB Expression Targets |
|  | NGF -> AP-1/TP53/MYC Expression Targets |
|  | NGF -> FOXO/MYCN/ELK-SRF Expression Targets |
|  | BDNF Expression Targets |
|  | NTF3 Expression Targets |
|  | NTF4 Expression Targets |
|  | NGF -> CREB/CEBPB/MEF2A Expression Targets |
|  | NOTCH -> NF-kB Signaling |
|  | DLL4 Expression Targets |
|  | ADAM17 Expression Targets |
|  | JAG1 Expression Targets |
|  | DLL1 Expression Targets |
|  | NOTCH Expression Targets |
|  | Adenosine Expression Targets |
|  | PDGFB -> AP-1/CREB/MYC Expression Target |
|  | PDGFB -> FOXO3A Expression Target |
|  | PDGFC -> CREB Expression Target |
|  | PDGFD -> AP-1 Expression Targets |
|  | PDGF -> AP-1/CREB/CREBBP/MYC Expression Targets |
|  | PDGF -> FOXO3A Expression Targets |
|  | KIT -> MITF Signaling |
|  | PDGFR -> AP-1/MYC Signaling |
|  | PDGFR -> FOXO3A Signaling |
|  | AVP/Gs -> CREB/ELK/SRF/AP-1/EGR Expression Targets |
|  | NTS Expression Targets |
|  | AVP/Gs -> MEF/MYOD/NFATC/MYOG Expression Targets |
|  | NPY Expression Targets |
|  | AVP/Gq -> MEF/MYOD/NFATC/MYOG Expression Targets |
|  | AVP/Gq -> CREB/ELK/SRF/AP-1/EGR Expression Targets |
|  | PRL/GHR -> NF/kB/ELK/SRF/MYC Expression Targets |
|  | PDCD1 -> ATF/CREB/CREBBP Expression Targets |
|  | PDCD1 -> NF-kB Expression Targets |
|  | PDCD1 -> NFATC Expression Targets |
|  | PDCD1 -> AP-1 Expression Targets |
|  | GUCYC2 Signaling |
|  | AngiopoietinR -> AP-1 Signaling |
|  | AngiopoietinR -> FOXO Signaling |
|  | GDNF -> HSF1 Signaling |
|  | EphrinB -> MAPK/JUN/FOS Signaling |
|  | NTRK -> FOXO/MYCN Signaling |
|  | NTRK -> AP-1/CREB/ELK/SRF/MYC/SMAD3/TP53 Signaling |
|  | GAS6 -> AP-1/CREB Expression Targets |
|  | FIGF -> AP-1 Expression Target |
|  | FIGF -> NCOR2 Expression Target |
|  | KITLG -> AP-1/CREB/CREBBP/MYC Expression Targets |
|  | CSF1 -> FOXO3A Expression Targets |
|  | GDNF -> HSF1 Expression Targets |
|  | CSF1 -> AP-1/CREB/CREBBP/MYC Expression Targets |
|  | PGF -> AP-1/CREB/CREBBP/MYC Expression Targets |
|  | Lysophosphatidic Acid/LPARs Signaling |
|  | Ras-GRF Regulation Signaling |
|  | Androgen Receptor non-Genomic Signaling |
|  | FOXO1 Signaling |
|  | MERTK Signaling |
|  | Ras Signaling |
|  | TGF-beta Signaling |
|  | AXL Receptor Tyrosine Kinase Signaling |
|  | PI3K/RAC1 Signaling |
|  | mTOR Signaling |
|  | TNF-alpha/TNFRSF1B Signaling |
|  | KRAS Signaling |
|  | VEGF Signaling |
|  | Estrogens/ESR1 non-Genomic Signaling |
|  | EGFR Signaling |
|  | HRAS Signaling |
|  | ERK/MAPK Canonical Signaling |
|  | Hedgehog Family Signaling |
|  | T-Cell Receptor -> NF-kB Signaling |
|  | TGFA -> FOXO3A Expression Targets |
|  | TGFA -> TP53 Expression Targets |
|  | TGFA -> HIF1A Expression Targets |
|  | TGFA -> MEF/MYOD/NFATC Expression Targets |
|  | TGFA/AP-1/ATF Expression Targets |
|  | TGFA -> CREB/CREBBP/ELK-SRF/MYC Expression Targets |
|  | CD40LG -> NF-kB/ELK/SRF -> CREB/NFATC Expression Targets |
|  | TNFSF13B Expression Targets |
|  | TNFRSF5/13B -> NFATC1 Signaling |
|  | Thrombopoietin -> SPI1 Expression Targets |
|  | Thrombopoietin -> AP-1/CREB/CREBBP/MYC Expression Targets |
|  | Thrombopoietin -> FOXO3A Expression Target |
|  | TLR1 -> 2/6 Expression Targets |
|  | TLR1/2/6 -> NF-kB Signaling |
|  | ROS in Neutrophil Mediated Cell Damage |
|  | ROS in Triggering Vascular Inflammation |
|  | Glutamate Mediated Neurotoxicity |
|  | ROS and RNS in Vasoconstriction and Vasodilation Regulation |
|  | Ca2+ Cytosolic Overload |
|  | ROS in Angiotensin Mediated Cardiovascular Remodeling and Hypertrophy |
|  | PLAU -> ELK-SRF/AP-1 Expression Targets |
|  | VEGFC -> CTNNB Expression Target |
|  | VEGFA -> FOXO3A Expression Targets |
|  | VEGFA -> AP-1/CREBBP/MYC Expression Targets |
|  | VEGFA -> CTNNB/CTNND Expression Targets |
|  | VEGFA -> NCOR2 Expression Target |
|  | VEGFR -> CTNNB Signaling |
|  | VEGFR -> AP-1/CREB/MYC Signaling |
|  | VEGFR -> FOXO3A Signaling |
|  | n |
|  | ace2 |
|  | Model of PPARG signaling in psoriasis |
|  | Model of PPARG related pathways in psoriasis (short version) |
|  | 3\_Increased proliferation of sebocytes in acne vulgaris |
|  | 2\_Interleukin-17 and interleukin-22 signaling in psoriasis |
|  | 1\_The onset of atopic dermatitis |
|  | 2\_Osteoblast-mediated bone demineralization |
|  | 1\_Formation of cysts in polycystic kidney disease |
|  | 2\_The role of endothelial cells in pyelonephritis |
|  | 3\_Mesangial cell dysfunction in glomerulonephritis |
|  | 1\_Local estrogen production stimulates ectopic endometrium proliferation |
|  | 3\_Enhanced angiogenesis in endometriosis |
|  | 2\_Impaired steroidogenesis in PCOS |
|  | 3\_Role of neurotrophic factor deprivation in retinal ganglion cell death |
|  | 2\_Pathogens stimulate mucins expression in the middle ear |
|  | 3\_Arterial wall inflammation and calcification in atherosclerosis |
|  | 2\_Oxidized LDL causes endothelial dysfunction in atherosclerosis |
|  | 1\_Endothelial cells stimulate vasoconstriction and thrombosis in pulmonary hypertension |
|  | 2\_Cardiomyocyte hypertrophy |
|  | 4\_Endothelial cells repress vasoconstriction in hypertension |
|  | 2\_The intense proliferation and contractile activity of smooth muscle cells in pulmonary hypertension |
|  | 1\_Cigarette smoke causes dysfunction and production of inflammatory mediators in alveolar epithelial cells |
|  | 3\_Role of alveolar macrophages in pulmonary tissue destruction in COPD |
|  | 1\_Th2 cell response in asthma |
|  | 3\_1\_Mucus accumulation in asthma |
|  | 2\_1\_Eosinophilia and neutrophilia in asthma: eosinophil migration and activation |
|  | 2\_2\_Eosinophilia and neutrophilia in asthma: eosinophil apoptosis |
|  | 4\_2\_Airway smooth muscle cells proliferation and airway tissue remodeling |
|  | 4\_Mucin hyperproduction in goblet and mucous cells in COPD |
|  | 2\_Intestine epithelial cells drive immunological responses in ulcerative colitis |
|  | 1\_Triglyceride storage in NAFLD |
|  | 1\_CD4+ T cell and epithelial cell response in celiac disease |
|  | 2\_CD8+ T-cell response and mucosal damage in celiac disease |
|  | 4\_Tax-induced disruption of the G1/S transition in HTLV-1 infected T-cell |
|  | 2\_HIV - induced depletion of CD4+ T-cells |
|  | 3\_1\_Beta-cell proliferation and compensation: proliferation |
|  | 1\_2\_Insulin synthesis and secretion: insulin secretion |
|  | 1\_1\_Insulin synthesis and secretion: insulin processing and storage |
|  | 3\_3\_Beta-cell proliferation and compensation: recovery |
|  | 4\_2\_Insulin resistance: inflammation-related insulin resistance |
|  | 4\_1\_Insulin resistance: insulin signaling |
|  | 1\_Antigen-presenting cells promote islet dysfunction and immune system activation in T1D |
|  | 1\_Microangiopathy in diabetic neuropathy |
|  | 2\_Neuron and Schwann cell death |
|  | 1\_Glutamate and calcium excitotoxicity of motor neurons in ALS |
|  | 1\_1\_Glutamate overload enforced prostaglandins and NO synthesis in neurons |
|  | 2\_1\_General pathways of thyroid hormone signaling |
|  | Model of PPARG signaling in psoriais (tested) |
|  | DIPG non histone mutations |
|  | DIPG overview\_2 |
|  | HRAS->RAF->MAPK1/3 |
|  | Interleukin-17 and interleukin-22 signaling in psoriasis |

---

|  |  |
| --- | --- |
| Source | curated |
|  | Biopax |

---

|  |  |
| --- | --- |
| MedScan ID | 1810341 |

---

|  |  |
| --- | --- |
| Alias | 3,4,5-PtdIns-P3 |
|  | 3,4,5-phosphatidylinositolphosphate |
|  | phosphatidylinositol-trisphosphate |
|  | dipalmitoyl phosphoinositol |
|  | 3,4,5-phosphatidylinositol trisphosphate |
|  | 3,4,5-PtdIns-P3s |
|  | phosphatidylinositol-triphosphate |
|  | phosphatidylinositol 3,4,5-P3 |
|  | phosphatidylinositol-3,4,5-phosphate |
|  | 3,4,5-PtdInsP |
|  | PIP(3)s |
|  | 3,4,5-phosphatidylinositol triphosphates |
|  | phosphatidylinositol 3,4,5-trisphosphate |
|  | phosphatidylinositol-3,4,5-phosphates |
|  | phosphatidylinositol 3',4',5' trisphosphate |
|  | phosphatidyl (3,4,5) inositol triphosphates |
|  | phosphatidylinositoltrisphosphates |
|  | phosphatidylinositoltriphosphate |
|  | 3,4,5-phosphatidylinositol trisphosphates |
|  | phosphatidylinositol 3,4,5-trisphosphates |
|  | 3,4,5-PtdInsP3 |
|  | PtdIns 3,4,5-P |
|  | phosphoinositide-3,4,5-triphosphate |
|  | 3,4,5-phosphatidylinositol P3 |
|  | phosphatidylinositoltriphosphates |
|  | 3,4,5-phosphatidylinositoltriphosphates |
|  | PI(3,4,5)P3 |
|  | PtdIns 3,4,5-P3 |
|  | phosphatidylinositoltrisphosphate |
|  | D-myo-Phosphatidylinositol 3,4,5-trisphosphates |
|  | 3,4,5-phosphatidylinositol-phosphates |
|  | D-myo-PI(3,4,5)P3 |
|  | phosphatidyl (3,4,5) inositol triphosphate |
|  | 3,4,5-PtdIns-P |
|  | 3,4,5-phosphatidylinositolphosphates |
|  | 3,4,5-phosphatidylinositoltrisphosphate |
|  | PtdInsP3 |
|  | 3,4,5-PtdInsP3s |
|  | 3,4,5-phosphatidylinositol P3s |
|  | PtdInsP3s |
|  | phosphoinositide-3,4,5-trisphosphate |
|  | 3,4,5-phosphatidylinositol triphosphate |
|  | PIP3s |
|  | 3,4,5-PtdIns-Ps |
|  | 165689-82-7 |
|  | phosphatidylinositol-triphosphates |
|  | phosphatidylinositol 3,4,5-P3s |
|  | phosphatidylinositol-trisphosphates |
|  | D-myo-Phosphatidylinositol 3,4,5-trisphosphate |
|  | 3,4,5-phosphatidylinositoltrisphosphates |
|  | dipalmitoylphosphoinositol |
|  | PIP3 |
|  | 3,4,5-phosphatidylinositoltriphosphate |
|  | PtdIns 3,4,5-Ps |
|  | 3,4,5-PtdInsPs |
|  | PIP(3) |
|  | 3,4,5-phosphatidylinositol-phosphate |
|  | phosphatidylinositol 3,4,5-triphosphates |
|  | PtdIns 3,4,5-P3s |
|  | phosphatidylinositol 3,4,5-triphosphate |

---

|  |  |
| --- | --- |
| CAS ID | 165689-82-7 |

---

|  |  |
| --- | --- |
| Reaxys ID | 7247590 |

---

|  |  |
| --- | --- |
| HMDB ID | HMDB04249 |

---

|  |  |
| --- | --- |
| WTF | false |
|  | true |

---

|  |  |
| --- | --- |
| ABCDWeb | http://www.abcd.com/Search/wewedwe |

---
